# Phosphodiesterase 4D Gene Modifies the Functional Network of Patients With Mild Cognitive Impairment and Alzheimer’s Disease

**DOI:** 10.3389/fgene.2020.00890

**Published:** 2020-08-06

**Authors:** Jie Xiang, Xin Wang, Yuan Gao, Ting Li, Rui Cao, Ting Yan, Yunxiao Ma, Yan Niu, Jiayue Xue, Bin Wang

**Affiliations:** ^1^College of Information and Computer, Taiyuan University of Technology, Taiyuan, China; ^2^Translational Medicine Research Center, Shanxi Medical University, Taiyuan, China

**Keywords:** PDE4D, fMRI, functional network, Alzheimer’s disease, graph theory

## Abstract

Alzheimer’s disease (AD) is a progressive neurodegenerative disorder that is affected by several genetic variants. It has been demonstrated that genetic variants affect brain organization and function. In this study, using whole genome-wide association studies (GWAS), we analyzed the functional magnetic resonance imaging and genetic data from the Alzheimer’s Disease Neuroimaging Initiative dataset (ADNI) dataset and identified genetic variants associated with the topology of the functional brain network http://www.adni-info.org. We found three novel loci (rs2409627, rs9647533, and rs11955845) in an intron of the phosphodiesterase 4D (PDE4D) gene that contribute to abnormalities in the topological organization of the functional network. In particular, compared to the wild-type genotype, the subjects carrying the PDE4D variants had altered network properties, including a significantly reduced clustering coefficient, small-worldness, global and local efficiency, a significantly enhanced path length and a normalized path length. In addition, we found that all global brain network attributes were affected by PDE4D variants to different extents as the disease progressed. Additionally, brain regions with alterations in nodal efficiency due to the variations in PDE4D were predominant in the limbic lobe, temporal lobe and frontal lobes. PDE4D has a great effect on memory consolidation and cognition through long-term potentiation (LTP) effects and/or the promotion of inflammatory effects. PDE4D variants might be a main reasons underlyling for the abnormal topological properties and cognitive impairment. Furthermore, we speculated that PDE4D is a risk factor for neural degenerative diseases and provided important clues for the earlier detection and therapeutic intervention for AD.

## Introduction

Alzheimer’s disease (AD) is a common, progressive, lethal neurodegenerative disorder. No preventative or curative treatment exists for this disease, which carries high emotional and economic costs (Heckman et al., 2015). The clinical phase of AD is marked by selective neuronal death, synaptic loss, and neurotransmitter loss (Holtzman et al., 2011), mainly because of genetic variance such as those of APOE and TAU (Ma et al., 1994; Petersen et al., 1995; Johnson and Jenkins, 1999; Ballatore et al., 2007). In addition, neuroinflammation has been proposed and accepted as a part of the process of neurodegeneration process in AD, based on both its occurrence and the genetic data that suggest a primary role of inflammation in the AD development (Calsolaro and Edison, 2016). Among these genetic variations, phosphodiesterase 4D (PDE4D) plays a crucial role in memory formation and consolidation through the cyclic nucleotide (cAMP and cGMP) signaling systems (Burgin et al., 2010). Zhang et al. (2014) also suggested that the loss of PDE4D function might attenuate neuroinflammation and confer neuroprotection in AD. Recently, phosphodiesterase inhibitors that enhance cAMP signaling have received increased attention as possible therapeutics for the treatment of AD (Burton et al., 2008; Zhang et al., 2014; Gurney et al., 2015).

Rapid progress in deciphering the biological mechanism of AD has arisen from the application of genotype-to-phenotype relationships (Selkoe, 2001). Recent genome-wide association studies (GWAS) on neuroimaging phenotypes have identified several genes and their variants associated with AD, such as APOE, TAU, TOMM40, ABCA7, CLU, CR1, CD33, CD2AP, EPHA1, BIN1, PICALM, MS4A (Bertram et al., 2008; Denise et al., 2009; Jean-Charles et al., 2009; Potkin et al., 2009; Hollingworth et al., 2011; Naj et al., 2011; Bettens et al., 2013; Lambert et al., 2013), and other novel genes, such as ANTXR2, OR5L1, IGF1, ZDHHC12, ENDOG and JAK1 (Elsheikh et al., 2020). In these genes, single nucleotide polymorphism (SNP) variants affect AD risk (Braskie et al., 2011; Hibar et al., 2015; Farfel et al., 2016; Gaiteri et al., 2016). The identification of SNPs has begun to create a broader picture of the genes and pathways involved in AD risk (Carlos et al., 2012; Jin et al., 2012; Jaehong et al., 2013; Peer-Hendrik et al., 2014). The study of considering multiple genes, multiple phenotypes, and the interactions between them has become an inevitable trend to gain insight into mechanisms in AD.

Neuroimaging techniques advance the study of genetic variation influence on brain structure and function (Glahn et al., 2007). Importantly, these neuroimaging phenotypes may be closely associated with the underlying biological etiology of the disease, making it easier to identify underlying genes (Grundman et al., 2002; Almasy et al., 2008; Potkin et al., 2009). Recently, graph theory and the topologic properties of the brain network have been advantageous for quantitatively evaluating functional abnormalities of AD in the whole brain (Tijms et al., 2013; Hugenschmidt et al., 2014). The functional brain networks were demonstrated to provide more direct association with the phenotype of AD (Sanz-Arigita et al., 2010; Tijms et al., 2012; De et al., 2014). For example, numerous studies have found a global decrease in functional connectivity, in which network efficiencies are decreased and the minimum path is increased in AD (Sanz-Arigita et al., 2010; Tijms et al., 2012). There was a decreased small-worldness value in AD and mild cognitive impairment (MCI) (De et al., 2014). Of note, previous studies have mostly found that the ApoE genotypeodulates brain network properties, especially in AD patients (Zhao et al., 2012; Wang et al., 2015). Another study also found that significant associations between the change in structural brain connectivity defined by tractography and genes (Elsheikh et al., 2020). However, the genetic variants associated with the topology of the functional brain network on the basis of whole genome-wide analysis and its pathological alterations in AD are largely unknown.

To the best of our knowledge, this is the first genome-wide study analyzing the association between genetic variance and functional brain network topology. We present an imaging genetics framework that employs whole-genome and whole-brain strategies to systematically evaluate genetic effects on brain imaging phenotypes and identify genetic risk variants in AD. The study was performed with resting-state functional magnetic resonance imaging (fMRI) and genetic data from the Alzheimer’s Disease Neuroimaging Initiative (ADNI) database. The SNP variances in PDE4D were found to affect the functional networks, which opens the possibility of exploring such directional functional relationships between gene expression and brain activity in human models.

## Materials and Subjects

### The ADNI Database and Subjects

The participants who had resting-state fMRI data, T1-weighted data and genetic data were selected from the ADNI database. The subjects provided written informed consent at the time of enrollment for imaging and completed questionnaires approved by the institutional review board (IRB) of each participating site, including Albany Medical College and Banner Alzheimer’s Institute (Mueller et al., 2005). These participants were assessed using a standardized clinical evaluation protocol, including a medical history interview, a neurologic examination, and a battery of neuropsychological tests. Neuropsychological tests included the Functional Assessment Questionnaire (FAQ) (Pfeffer et al., 1982), Geriatric Depression Scale (GDS) (Sheik and Yesavage, 1986), Mini-Mental State Examination (MMSE) (Folstein et al., 1975), and Montreal Cognitive Assessment (MoCA) (Nasreddine et al., 2010).

### Imaging and Gene Data Acquisition

The fMRI data were collected according to the ADNI acquisition protocol using three Tesla (3T) scanners (Philips Medical Systems). The resting-state fMRI data of each subject consisted of 140 functional volumes and were acquired using the following parameters: repetition time (TR) = 3000 ms; echo time (TE) = 30 ms; flip angle = 80°; slice thickness = 3.313 mm; and 48 slices. The T1-weighted images were acquired by the following parameters using a magnetization-prepared rapid gradient-echo (MPRAGE) sequence: 170 sagittal slices; slice thickness = 1.2 mm; repetition time (TR) = 6.80 ms; echo time (TE) = 3.16 ms; flip angle = 8.0°; field of view (FOV) = 256 × 256 mm^2^; and acquisition matrix = 256 × 256. All subjects were instructed to keep their eyes closed but not fall asleep, relax their minds, and move as little as possible during the data acquisition. The genetic data were acquired by using the Illumina Omni 2.5M (WGS Platform) and Illumina HumanOmniExpress BeadChip in the ADNI database, which include 3.7 million SNPs and 730525 SNPs, respectively.

### Imaging Analysis

The data preprocessing was conducted using Statistical Parametric Mapping (SPM8) software^1^ and Data Processing Assistant for Resting-State fMRI (DPARSF). For each run, the first 10 time points were removed and then realigned to the first volume to correct for head motion, which did not exceed 2.0 mm of displacement or 2.0° of rotation in any direction, in any subject. Subsequently, the functional images were spatially normalized to the standard Montreal Neurological Institute (MNI) template and resampled to a voxel size of 3 × 3 × 3 mm^3^. The waveform of each image was then processed for further smoothing and nuisance covariate regression. Mean time series were subsequently extracted from the 90 automated anatomical labeled (AAL) nodes for each subject and paradigm to minimize the effects of the length of the time series on the derived reliability estimates of the graph properties.

### Construction of the Functional Brain Network

After data preprocessing, regions based on the AAL nodes^2^ (Kelly et al., 2008) were extracted per subject. The fMRI time series was computed in each of the 90 regions by averaging all voxels within each region at each time point in the time series, resulting in 130 time points for each of the 90 anatomical regions. Then, these time series were used to construct a 90-node whole-brain functional connectivity network by calculating the Pearson correlation coefficient in the residual time courses between each pair of regions of interest (ROIs), and 90 × 90 correlation matrices were obtained for each subject. Finally, each correlation matrix was repeatedly thresholded into a binarized matrix with a wide range of sparsity (10% to 40%) with intervals of 0.01. Through this thresholding, unweighted graphs were obtained in which the nodes represent the brain regions and the edges represent the functional relationships between brain regions. Further network analyses were based on the 90 × 90 binarized matrices of each subject.

### Analysis of Network Properties

The GRETNA toolbox^3^, implemented in MATLAB (MathWorks, Inc.), was used for network analyses. The network architecture was investigated at both the global and regional levels in the constructed resting-state functional brain networks. The following seven network metrics were adopted to characterize the global topological organization of the brain networks: clustering coefficient, Cp; characteristic path length, Lp; normalized clustering coefficient, γ; normalized shortest path length, λ; small-worldness, σ, σ = γ/λ; global efficiency, Eg; and local efficiency, Eloc. Here, we provide an overview of the definitions and brief interpretations of the parameters (Supplementary Table 1).

### Quality Control of Genetics Data

Originally, 143 subjects were selected from the ADNI2 database. The following quality control (QC) was performed on the genetic data using the PLINK software package^4^, release v1.06. SNPs were excluded from the imaging genetics analysis if they did not meet any of the following criteria: (1) call rate per SNP ≥ 90%, (2) minor allele frequency (MAF) ≥ 5%, and (3) Hardy-Weinberg equilibrium test of *p* ≤ 10^–6^ using only healthy control (NC) subjects. The overall genotyping rate for the remaining dataset was over 99.5%. In addition, participants were excluded from the analysis if any of the following criteria were not satisfied: (1) call rate per participant ≥90% (one participant was excluded); (2) gender check (two participants were excluded); and (3) identity check (three sibling pairs were identified with PI_HAT over 0.5; one participant from each pair was randomly selected and excluded). After the QC procedure, 137 out of 143 participants and 2,406,870 SNPs remained in the analysis.

### GWAS Analysis

GWAS analysis on selected brain network properties of 137 subjects was completed using the quality-controlled SNP data, as follows. Mean global attributes derived from 90 nodes constituted the trait of interest. Genotype data were analyzed using an additive model, with odds ratios (ORs) or regression coefficients expressing the effect of each copy of the reference allele. Continuous measures with skewed distributions were log transformed. Adjustments were made for baseline age, sex, and education prior to any of the GWAS analyses (Risacher et al., 2009). Using PLINK, each GWAS analysis calculated the main effects of all SNPs on the functional brain network properties (Eg, Eloc, Lp, Cp, λ, γ, σ). Manhattan and LocusTrack plots were used to visualize the GWAS results. All association results surviving the significance threshold of *p* < 10^–7^ were saved and prepared for additional pattern analysis. The less stringent threshold (blue line for *p* < 10^–6^) was set for the evaluation of evaluating to help guide the refined analyses.

### Statistical Analysis

Statistical analyses were performed using SPSS version 20.0 (SPSS Inc., Chicago, IL). Analysis of variance (ANOVA) was used to test for group differences in age and education. The difference of gender was explored using chi-squared tests.

Analyses of covariance (ANCOVAs, corrected for age, gender and education-year corrected) were used to test for group differences in network topology metrics. First, we performed the one-way ANCOVAs and only considered the effects of genotype (variant versus wild-type) on brain network properties. Then, we performed the two-way ANCOVAs with the factors of genotype (variant versus wild-type) and group (NC, MCI and AD). For nodal efficiency, Bonferroni correction was used for multiple comparisons. In addition, we used partial correlations controlled for age, gender, and education year to examine the relationship between properties and clinical performance.

## Results

### Sample Characteristics After QC

After QC of the original data, 137 out of 143 ADNI participants remained in the present study, including 42 NC, 65 MCI, and 30 AD patients. Table 1 shows the demographic information of the sample analyzed for both characteristics and neuropsychological test studies.

**TABLE 1:** Results of demographic characteristics and neuropsychological test.

**Characteristic**	**NC (*n* = 42)**	**MCI (*n* = 65)**	**AD (*n* = 30)**	**Test statistic**	***P*-value**
Age (years)	73.42 ± 5.54	70.73 ± 7.66	72.54 ± 7.48	*F* = 3.348	0.04
Gender (male/female)	19/23	31/34	13/17	*x*2 = 0.347	0.841
Education level (years)	16.12 ± 2.34	15.82 ± 2.76	16.28 ± 2.93	*F* = 0.232	0.794
FAQ	0.09 ± 0.37	2.65 ± 3.70	14.63 ± 7.61	*F* = 35.776	<0.001abc
GDS	0.62 ± 0.96	1.48 ± 1.39	1.57 ± 1.10	*F* = 6.393	0.002a
MMSE	28.76 ± 1.32	28.12 ± 1.58	22.77 ± 2.57	*F* = 56.785	<0.001bc
MOCA	22.48 ± 1.36	20.78 ± 0.81	23 ± 1.53	*F* = 1.319	0.272

*^*A*^Values are given as mean ± SD. ^*B*^NC:normal control; MCI: amnestic mild cognitive impairment; AD: Alzheimer’s disease; FAQ: Functional Assessment Questionnaire Total Score; GDS: Geriatric Depression Scale Total Score; MMSE: Mini-Mental State Examination Total Score; MOCA: Montreal Cognitive Assessment Total Score. ^*a*^normal control group and MCI group showed significant differences (*P* < 0.001). ^*b*^normal control group and AD group showed significant differences (*P* < 0.001). ^*c*^MCI group and AD group showed significant differences (*P* < 0.001).*

### GWAS Analysis of Functional Network Properties

**FIGURE 1:**
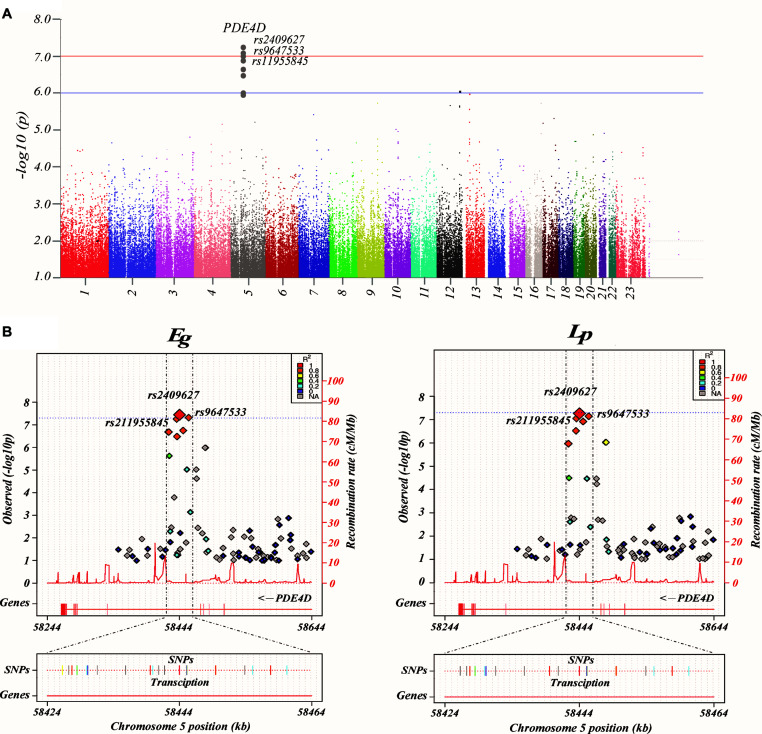
Common genetic variants associated with brain networks. **(A)** Manhattan plot presentation of the genome-wide association study (GWAS). The parameters examined in this analysis are the brain network properties, which were calculated using MATLAB and adjusted for age, sex, and education. Shown on the left of the Manhattan plot are the *p*-values [-log10 (observed *p*-value)] from the GWAS analysis. The horizontal lines display the cutoffs for two significance levels: blue line for *p* < 10^–6^ and red line for *p* < 10^–7^. **(B)** Regional association and functional annotation of previously unknown genome-wide significant loci. Regional association plots for the 3 significant loci of the GWAS results on brain network properties (GENCODE version 19). Plots were generated using LocusTrack software (http://gump.qimr.edu.au/general/gabrieC/LocusTrack/).

By using GWAS (*p* < 10^–7^ significance level), three SNPs (rs2409627, rs9647533, rs11955845) that belonged to the PDE4D (Table 2) had associations with the functional network properties, including Eg, Eloc, Lp, Cp, λ, γ, σ (Figure 1A). The SNPs on chr. 12 and 13 also showed differences though (*p* < 10^–6^ significance level), they were not formally introduced in this paper. First, strictly speaking, the significance level did not meet the threshold criteria (*p* < 10^–7^). Second, as there were no continuous results on chr. 12 and 13, which shows that they may have happened by chance. Last, they are not located in exons, and their specific functions are thus unclear.

**TABLE 2:** Top quantitative trait (brain network properties) loci ranked by the total number of associations at the significance level of *p* < 10^–7^.

**RsID**	**Chr**	**Pos**	**Gene**	**Allele 1**	**Allele 2**	**Freq**	***P*-value**
rs2409627	5	58444322	PDE4D	C	T	0.1282	2.74E-08
rs9647533	5	58458117	PDE4D	C	T	0.1325	4.15E-08
Rs11955845	5	58439884	PDE4D	G	A	0.1356	4.66E-08

*Relevant information about top-ranked SNPs and their respective genes is shown in this table, including SNP, chromosome (Chr), Pos (position), Gene, Allele, Freq (allele frequency is given with reference to Allele 1) and *P*-value.*

Among these seven network properties, Eg and Lp had identified to have stronger associations with the SNPs of PDE4D than the rest of the properties as determined by the loci results. It should be noted that the other variants of PDE4D also showed a level of association with Eg and Lp (Figure 1B).

### Global Properties of the Functional Brain Networks

**FIGURE 2:**
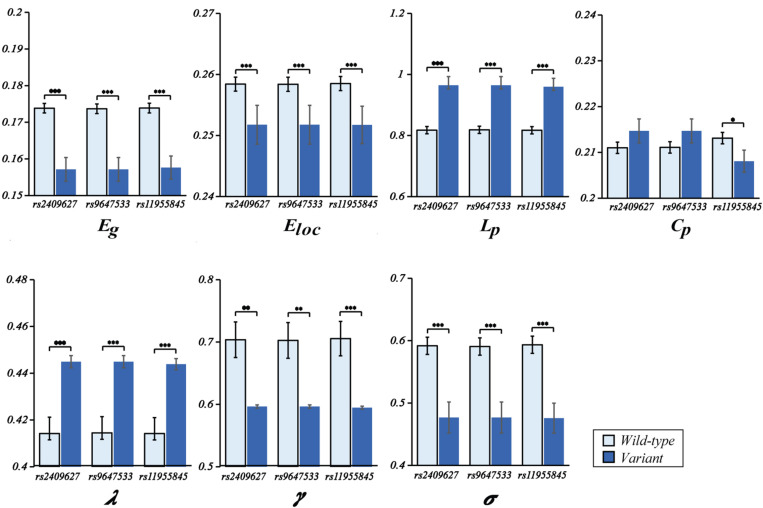
One-way ANOVA of mean (SD) brain network properties between wild-type and the variants. ANOVAs were applied to examine the effects of variants on brain network properties. All the analyses included age, sex, and education as covariates (*P < 0.05, **P < 0.01, ***P < 0.001). There were 104 subjects with PDE4D wild-type and 33 subjects with PDE4D variants, and three SNPs had the same number.

**FIGURE 3:**
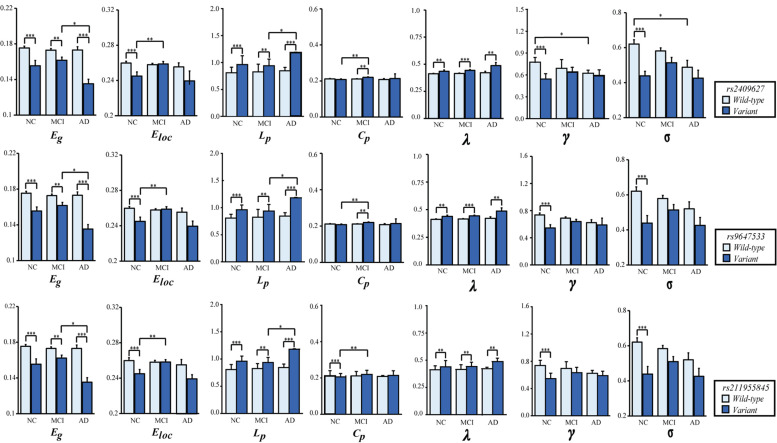
Two-way ANOVA was applied to examine the effects of genotype and group on the mean (SD) brain network properties. All the analyses included age, sex, and education as covariates. The *p*-values for the main effect of group and the main effect of SNP are shown in each plot (*P < 0.05, **P < 0.01, ***P < 0.001). The number of subjects with different genotypes in each diagnostic group is detailed in Supplementary Table 6.

As shown in Figure 2, we observed that the 3 SNPs showed the same trends in different brain network properties. Compared with wild-type genotype, the PDE4D variants had significantly lower Eg, Eloc, Cp, γ and σ values (*F* > 4.142, *p* < 0.043) and significantly higher Lp and λ values (*F* > 28.364, *p* < 3.053E-07). As shown in Figure 3, we observed that the 3 SNPs showed different brain network properties among these diagnostic groups. In addition to the significant genetic effect of these properties, we also found that the interaction effect of group and genotype was significant for Eloc (rs2409627: *F* = 5.346, *p* = 0.006; rs9647533: *F* = 5.36, *p* = 0.006; rs11955845: *F* = 4.92, *p* = 0.009) and Cp (rs2409627: *F* = 3.48, *p* = 0.034; rs9647533: *F* = 3.42, *p* = 0.036; rs11955845: *F* = 4.98, *p* = 0.009), but not for the other properties. Pairwise comparisons indicated that the NC group with PDE4D variants had significant differences in network properties compared to the wild-type genotype (*F* > 7.053, *p* < 0.009) except for the Cp. The MCI patients carrying PDE4D variants showed significantly lower Eg values but higher Lp, Cp and λ values than the non-carrier patients (*F* > 7.119, *p* < 0.009). The Lp and λ values were significantly enhanced, and the Eg value was reduced in AD patients with PDE4D variants compared to those with the wild-type genotype (*F* > 7.505, *p* < 0.007).

In regard to the group differences for the subjects with the PDE4D variants, significant reductions of Eg values were found in the AD group versus the MCI group (*p* < 0.05, Bonferroni corrected); the Eloc values were significantly higher in the MCI group than in the NC group (*p* < 0.05, Bonferroni corrected); Lp values were significantly increased in the AD group compared with the MCI group (*p* < 0.05, Bonferroni corrected); and the Cp values were obviously enhanced in the MCI group compared with the NC group (*p* < 0.05, Bonferroni corrected). On the other hand, we observed significant reductions in γ and σ in the AD patients who did not have the PDE4D variants compared to the NCs (*p* < 0.05, Bonferroni corrected). However, no group differences were found for other properties and the subjects with the wild genotype.

### Nodal Properties of the Functional Brain Networks

**FIGURE 4:**
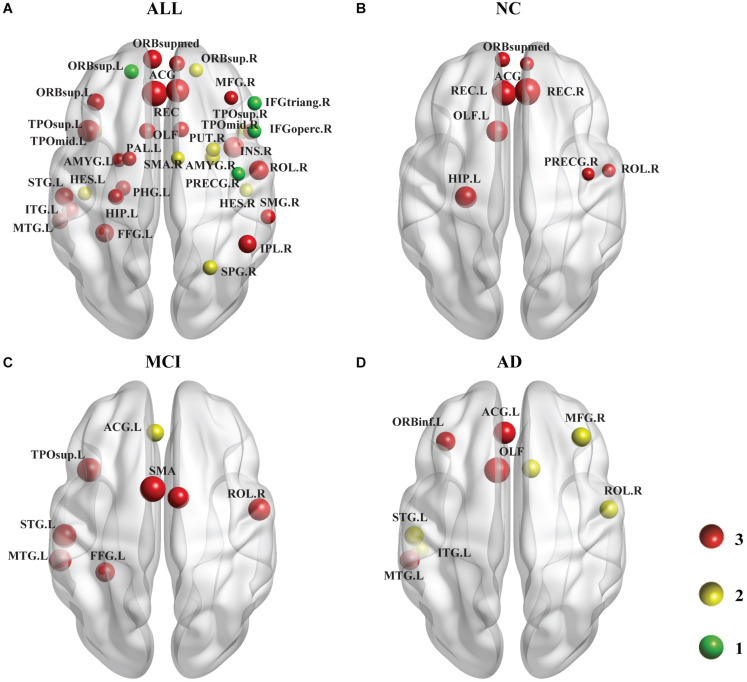
Following the analysis of the nodal efficiency (regional), PDE4D variant-related alterations in regional efficiency are shown in this plot. Among these regions, the red ball suggested that all 3 SNPs obtained from the GWAS analysis had obvious associations with this region, the yellow color represents 2 SNPs, and the green color represents 1 SNP. The size of the ball is determined by –log10 (observed *p*-value). **(A)** shows the results of a comparison involving all 137 subjects (i.e., across all the diagnostic groups), which are displayed at a threshold of *p* < 0.001 (Bonferroni corrected). **(B–D)** show the results of comparisons at a threshold of *p* < 0.05 (Bonferroni corrected) representing the three groups. The areas of the brain that are not marked left and right are bilateral. Age, sex, and education were included as covariates in all comparisons.

As shown in Figure 4, we identified the brain regions in which the nodal efficiency was significantly reduced by the PDE4D variants. For all subjects (Figure 4A), a number of regions were significantly affected by the variants (*p* < 0.001, Bonferroni corrected), including the temporal lobe (e.g., PUT.R, PAL.L, bilateral HES, STG.L, bilateral TPOsup, MTG.L, bilateral TPOmid, ITG.L), frontal lobe (e.g., PRECG.R, bilateral ORBsup, MFG.R, IFGoperc.R, IFGtriang.R, ORBinf.L, ROL.R, SMA.R, bilateral ORBsupmed, bilateral REC, bilateral ACG), parietal lobe (FFG.L, SPG.R, IPL.R, IPL.R, SMG.R), limbic lobe (bilateral OLF, HIP.L, PHG.L, bilateral AMYG), and insula (INS.R). We further identified the brain regions showing significant differences in each group (*p* < 0.05, Bonferroni corrected). Generally, the NC group had more abnormal nodes than the MCI and AD groups. The NC group showed significant regions in both the frontal (e.g., PRECG.R, ROL.R, bilateral ORBsupmed, bilateral REC, bilateral ACG) and limbic (bilateral OLF, HIP. L) (Figure 4B) lobes. The MCI patients exhibited significant differences in both the temporal lobes (e.g., FFG.L, STG.L, TPOsup.L, MTG.L) and frontal lobes (e.g., ROL.R, bilateral SMA, ACG.L) (Figure 4C). The AD patients showed significant differences in several brain regions, including the temporal lobe (e.g., STG.L, MTG.L, ITG.L), frontal lobes (e.g., MFG.R, ORBinf.L, ROL.R, ACG.L), and limbic lobe (bilateral OLF) (Figure 4D). The details of the brain regions in which the nodal efficiency was significantly reduced by these three SNP variants compared to the wild-type genotype are shown in Supplementary Tables 2–5.

### The Relationship Between PDE4D and Neuropsychological Variables

We did not find any significant differences in these neuropsychological scores between PDE4D variables.

## Discussion

Employing functional brain networks and whole-genome SNPs, we provided a methodological framework for systematically identifying the influences of genetic variants on the functional brain network. First, we confirmed the feasibility of a hypothesis-blind, multivariate approach to corroborate new putative disease-relevant genes (PDE4D) associated with known pathologic mechanisms. Second, we investigated the effects of PDE4D variants on the global topological organization of the brain network and found that all brain network attributes were affected by PDE4D variants to different extents, but the damage was more serious in the AD group. Third, alterations in nodal efficiency due to variations in PDE4D in various brain regions have been linked to AD, predominantly including the frontal, temporal and limbic lobes. These findings support the hypothesis that the PDE4D variants are evidence of cognitive impairment and help to fill the gap between the effects of genetic variants on brain networks.

### PDE4D and Cognitive Dysfunction in Alzheimer’s Disease

Through GWAS on network properties, we identified specific genes (PDE4D) correlated with the topological organization of the brain network in the normal controls and MCI and AD patients. Among the different PDE4 isoforms, PDE4D has been a prominent research focus due to accumulating evidence of its crucial role in cognitive processes, making it this enzyme a promising target for the development of therapeutic interventions in a variety of pathological conditions characterized by memory impairment, such as AD, stroke, and epilepsy (McLachlan et al., 2007; Richter et al., 2013; Bruno et al., 2014; Gurney et al., 2015; Ricciarelli and Fedele, 2015; Wang and Jiang-Ping, 2016). Recently, Blokland et al. (2012) suggested that PDE4D inhibitors may have utility for improving the symptoms of cognitive decline associated with neurodegenerative and psychiatric diseases. There are two potential neurobiological interpretations for this association. First, PDE4D loss of function might attenuate neuroinflammation and confer neuroprotection in AD. It has been well demonstrated that PDE4D hydrolyzes cAMP (Ricciarelli and Fedele, 2015) and then inhibits anti-inflammatory effects in the human brain (Richter et al., 2013; Zhang et al., 2014; Ricciarelli and Fedele, 2015). The ultimate effects of the complex inflammatory, ionic, and oxidative changes that occur in affected cortical regions are neurotic dystrophy, synaptic loss, shrinkage of neuronal perikarya, and selective neuronal loss, which then influence cognitive function (Dantzer et al., 2008; Harrison et al., 2011; Calsolaro and Edison, 2016). Presumably, these processes occur gradually over many years in the preclinical phase of AD and then continue during its clinical progression (Calsolaro and Edison, 2016). Second, a vast number of investigations have demonstrated that boosting long-term potentiation (LTP) by blocking PDE4D represents the molecular trigger to improve memory formation and consolidation (Ricciarelli and Fedele, 2015). As LTP is considered a correlate of learning and memory, many findings have suggested that age-related changes in the LTP level may also impact cognitive functions during aging (Rose et al., 2005; Villeda et al., 2011; Ricciarelli et al., 2017). Moreover, substantial evidence has shown that PDE4D inhibitors are able to rescue compromised LTP in different models of pathological conditions, including AD (Bliss and Collingridge, 1993; Ricciarelli and Fedele, 2015). A study of positron emission tomography imaging found that between 20 and 25% of patients diagnosed with AD do not have an amyloid burden (Gurney et al., 2015). At present, hypotheses regarding the pathological mechanism of AD include Aβ deposition, abnormal tau protein phosphorylation, and oxidative stress. However, none of the above hypotheses can explain all the pathological features of AD (Richter et al., 2013). The results of our study suggest that the PDE4D variant is indeed associated with abnormal expression of global functional brain networks. This is why we think that PDE4D inhibitors could represent a very promising cognitive-enhancing drug with great potential for the treatment of AD.

### Abnormal Network Topologies Associated With PDE4D Variants

In our study, the brains of all subjects who carried a PDE4D variant were characterized by significant differences in global brain network properties in all subjects. The lesser globally integrated networks and locally integrated networks are seen as a parallel for cognitive dysfunction. De et al. (2014) also implied that the lower global efficiency, longer Lp, weaker local efficiency and Cp network constituted the basis of cognitive impairment (Hugenschmidt et al., 2014; Zhou et al., 2016). It has been widely interpreted that the larger global efficiency and shorter Lp ensure global effective integrity or prompt transfers of information in brain networks (Lopes et al., 2017; Wang et al., 2017). Cp and local efficiency are measures of local network connectivity, and the subjects with the PDE4D variants had lower values, indicating that these subjects have weaker local information processing capacity. The above results show attenuated long-distance functional connections and decreased local functional connections in the brain network of subjects with the PDE4D variants, and these network properties were very similar to those found in previous studies examining brain networks in subjects showing cognitive impairment (Bassett and Sporns, 2017; Murphy and Fox, 2017; Rathore et al., 2017; Wang et al., 2018; Yan et al., 2018). This supports our hypothesis that PDE4D variants can be neurotoxic and even lead to cognitive impairment. A number of recent studies, similar to our results, have also suggested a link between inflammatory processes and MRI-detected anomalies in the brains of individuals with major depressive disorder (MDD), older adults with cognitive impairment and individuals with AD and schizophrenia (Dantzer et al., 2008; Eisenberger et al., 2010; Harrison et al., 2011; Frodl and Amico, 2014). Thus, our findings indicated that the disruption of the LTP effect and/or the inflammatory effect caused by the PDE4D variants might be the main reason for the abnormal topological properties and cognitive impairment.

Interestingly, we found that properties were more commonly affected by the PDE4D variants in the NC group, and more seriously affected in the AD group, as shown by Eg, Lp and λ. For the NC group, the PDE4D variants are potentially the main reason for the cognition disruption in older subjects, resulting in decreased Eg, Eloc, Cp and small-worldness and increased Lp and λ. Supporting our findings, several functional network studies have revealed decreased global and local efficiencies of brain networks in older people compared with younger adults (Greicius et al., 2009; Heuvel and Pol, 2010). We proposed that the LTP effect could explain the high susceptibility of the brains in the NC group. A previous study confirmed that the LTP effect is more susceptible to obstruction in the NC group than in the patient group (Mclachlan et al., 2007). This might be because the expression of the majority of the PDE4D isoforms was remarkably reduced in the AD hippocampus compared with the normal hippocampus (Mclachlan et al., 2007). Moreover, Calsolaro and Edison (2016) described that memory loss also occurs in normal elderly people and that brain cells are withered, while with the progression of AD, memory loss gradually accelerates, and the brain would triggers a more persistent inflammatory status with the progression of AD, leading to neuronal damage and neurodegeneration. These results also support that PDE4D is an accomplice, resulting in a longer-lasting inflammatory response. Thus, the difference is more obvious in the comparative analysis of the brain network attributes in the AD group.

### Regional Efficiency of PDE4D Variants

The regional efficiency in these brain networks was also found to be significantly decreased in the cortical regions, which were predominately located in the temporal lobe (e.g., bilateral HES, STG.L, bilateral TPOsup, MTG.L, bilateral TPOmid, ITG.L), frontal lobe (e.g., PRECG.R, bilateral ORBsup, MFG.R, ORBinf.L, ROL.R, SMA.R, bilateral ORBsupmed, bilateral REC, bilateral ACG), and limbic lobe (bilateral OLF, HIP.L, bilateral AMYG). These regions form circuits to coordinate movement, learning, memory and motivation, and altered circuits can lead to abnormal behavior and disease. For example, it has been demonstrated that hippocampal LTP is thought to serve as an elementary mechanism for the establishment of certain forms of explicit memory in the mammalian brain (Rose et al., 2005; Villeda et al., 2011; Ricciarelli et al., 2017). The regions in the temporal lobe involve linguistic integration, emotion, and semantic memory (Tijms et al., 2013). Moreover, the frontal regions are thought to be involved in the emotional, memory, and executive functions (Heuvel and Pol, 2010; Urs et al., 2015). In addition, inflammation in the medial frontal lobe and temporal lobe leads to significantly reduced cognition (Dantzer et al., 2008; Eisenberger et al., 2010; Harrison et al., 2011; Calsolaro and Edison, 2016). Thus, these regions are important for memory and cognition, and deficits in network efficiency can lead to abnormal behavior and disease.

We noticed that regional alterations showed different patterns in all diagnostic groups. The NC group had significant differences in the frontal lobe, the MCI group had significant differences in the temporal lobe, and the AD group had obvious differences in both the frontal and temporal lobes. Consistent with our results, Mirra and Gearing (1997) proposed that as AD progresses, the temporal neuropathologic lesions and inflammation are significantly enhanced. Neurobiology can explain this phenomenon. The hydrolytic capacity of PDE4D is directly proportional to the inflammatory effect (Gurney et al., 2015; Ricciarelli et al., 2017), and we thus propose that the hydrolytic capacity of the PDE4D variants are significantly differs from that of the wild-type in the regions affected by inflammation during AD. Some neuroimaging studies of AD have found increased frontal activity during certain cognitive tasks compared with MCI (Becker et al., 1996; Woodard et al., 1998; Jérémie et al., 2010). These increases in frontal activities have been interpreted as a compensatory reallocation or recruitment of cognitive resources (Wang et al., 2010). Indeed, genetic deletion of PDE4D displayed an improvement in spatial and recognition memory, and PDE4D inhibitors could thus represent a very promising cognitive-enhancing drug (Mclachlan et al., 2007; Gurney et al., 2015; Ricciarelli et al., 2017). In conclusion, PDE4D inhibitors have great potential for the treatment of AD in the early or preclinical stage (Richter et al., 2013; Heckman et al., 2015).

### Relationship Between Brain Network Properties and Cognitive Performance

A number of previous studies have clearly indicated a key role for PDE4D in hippocampus-dependent cognition (Mclachlan et al., 2007; Richter et al., 2013; Bruno et al., 2014; Gurney et al., 2015; Heckman et al., 2015; Ricciarelli et al., 2017). PDE4D is related to a variety of neurological diseases, such as AD, stroke, and epilepsy (Rose et al., 2005; Bales et al., 2010; Richter et al., 2013; Bruno et al., 2014; Wang and Jiang-Ping, 2016). Indeed, the facts have suggested that PDE4D inhibitors work on memory and cognitive enhancement as well as have neuroprotective and neurodegenerative properties in preclinical models (Ricciarelli and Fedele, 2015; Wang and Jiang-Ping, 2016; Prickaerts et al., 2017; Ricciarelli et al., 2017). However, we found no identified associations between cognitive performance and the PDE4D variants. These cognitive tests were mainly used for evaluating the overall cognitive levels of the AD patients and had low sensitivity for evaluating the differences due to the PDE4D variants.

## Limitations

The majority of analyses presented in this study focused on filling the gap between the effects of genetic variants on brain networks. However, due to the novel brain network analysis of this gene, there were some limitations in the present study. First, future studies could incorporate additional variables (e.g., clinical measures, other types of imaging and biomarkers) in the GWAS design to examine their effects and interactions with SNPs and/or target imaging phenotypes. Second, although the ADNI is such a large dataset, the fMRI data collection parameters of the subjects should be consistent, and there should be matched genetic data, which will lead to a small number of eligible subjects. Last, the characteristic influences of the PDE4D influences on the topologies of functional brain networks in patients with other brain neural disorder diseases (e.g., epilepsy, stroke) remain largely unknown. Additionally, the brain network mechanism of the cognitive impairment due to PDE4D, and the enhancement of PDE4D inhibitors need further study.

In conclusion, we herein confirmed a significant association between functional brain networks and genetic variants. These results suggest that PDE4D knockdown may offer a promising treatment for memory loss associated with cognitive impairment and/or AD. Furthermore, we speculated the PDE4D is a risk factor for the neural degenerative disease, and provided important clues for earlier detection and therapeutic intervention of AD.

## Data Availability Statement

Data collection for this article was obtained from the Alzheimer’s Disease Neuroimaging Initiative (ADNI) database (adni.loni.usc.edu). The ADNI unites researchers with study data as they work to define the progression of Alzheimer’s disease (AD). ADNI researchers collect, validate and utilize data, including serial magnetic resonance imaging (MRI), positron emission tomography (PET), genetics, cognitive tests, CSF and blood biomarkers as predictors of the disease to measure the progression of MCI and early AD.

## Author Contributions

JXi, XW, YG, and TL designed the study. XW, YG, and TL performed the experiments and data acquisition. XW, YG, TL, and YN performed the analysis and interpretation of the data. XW and YG wrote the draft of the manuscript and contributed to the manuscript revision. All authors approved the manuscript to be published and agreed on all aspects of the work.

## Conflict of Interest

The authors declare that the research was conducted in the absence of any commercial or financial relationships that could be construed as a potential conflict of interest.

## Footnotes

1 http://www.fil.ion.ucl.ac.uk/spm/

2 http://www.cyceron.fr/freeware/

3 http://www.nitrc.org/projects/gretna/

4 http://pngu.mgh.harvard.edu/~purcell/PLINK/
