# Supplementary material for: Phosphodiesterase 4D Gene Modifies the Functional Network of Patients With Mild Cognitive Impairment and Alzheimer’s Disease
Source: Front Genet. 2020 Aug 6;11:890. doi: 10.3389/fgene.2020.00890 (PMC7423997; doi:10.3389/fgene.2020.00890)
Supplement: Supplementary file 1 [file Data_Sheet_1.pdf]

Supplementary Material

Supplementary Figures and Tables

Supplementary Table 1. Formulations and descriptions of network topological properties applied in this work.

Network properties	Definitions	Measurement and interpretation
Global efficiency (*Eg*)	$Eg = \frac{1}{N(N-1)} \sum_{i,j\in N, i\neq j} \frac{1}{d_{ij}}$	*Eg* reflects the efficiency of the whole network information transmission.
Local efficiency (*Eloc*)	$Eloc = \frac{1}{N} \sum_{i\in N} E_g(i)$	*Eloc* is a measure of the mean local efficiency of the network.
Characteristic path length (*Lp*)	$Lp = \frac{1}{N(N-1)} \sum_{i,j\in N, i\neq j} d_{ij}$	N is the number of nodes in the network G. d_{ij} is the shortest path length between node i and j. *Lp* is the inverse relation of *Eg*.
Clustering coefficient (*Cp*)	$Cp = \frac{1}{N} \sum_{i\in N} \frac{\sum_{j,l} a_{ij} a_{jl} a_{li}}{k_i(k_i - 1)}$	*Cp* measures the extent of the local density of a network G with N nodes (N = 90 in this work). Here, k_i is the number of edges connecting to node i and a_{ij} is the edge between region i and j.
Normalized characteristic path length (λ)	$\lambda = Lp/L_{random}$	L_{random} represents the characteristic path length of the random network.
Normalized clustering coefficient (γ)	$\gamma = \mathrm{Cp}/\mathrm{C_{random}}$	C_{random} represents the clustering coefficient of the random network.
Small-worldness property (σ)	$\sigma = \gamma/\lambda$	σ is a measure of the small-worldness.
Nodal Efficiency (E_{nodal})	$E_{nodal}(i) = \frac{1}{(N-1)} \sum_{j\in G} \frac{1}{L_{i,j}}$	$E_{nodal}(i)$ measures how efficient an index node communicates with the other nodes.

Supplementary Table 2. Information on brain regions in which nodal efficiency was significantly reduced by PDE4D variants across all subjects.

ALL subjects		rs2409627		rs9647533		rs11955845	
Regions	abbr.	F	P	F	P	F	P
Precentral gyrus	PreCG.R	5.468	2.086E-02	7.746	6.313E-03	6.432	1.256E-02
Superior frontal gyrus, orbital part	ORBsup.L	8.641	3.878E-03	5.781	1.782E-02	6.546	1.182E-02
Superior frontal gyrus, orbital part	ORBsup.R	7.555	6.816E-03	6.643	1.124E-02	7.989	5.560E-03
Middle frontal gyrus	MFG.R	6.813	1.009E-02	7.068	8.984E-03	11.148	1.137E-03
Inferior frontal gyrus, opercular part	IFGoperc.R	6.249	1.364E-02	5.959	1.620E-02	7.412	7.498E-03
Inferior frontal gyrus, triangular part	IFGtriang.R	8.070	5.210E-03	5.922	1.652E-02	5.751	1.811E-02
Inferior frontal gyrus, orbital part	ORBinf.L	11.061	1.140E-03	14.509	2.273E-04	15.365	1.517E-04
Rolandic operculum	ROL.R	14.229	2.427E-04	17.993	4.558E-05	19.062	2.799E-05
Supplementary motor area	SMA.R	6.847	9.907E-03	7.695	6.479E-03	6.129	1.477E-02
Olfactory cortex	OLF.L	10.257	1.704E-03	11.067	1.186E-03	11.088	1.171E-03
Olfactory cortex	OLF.R	8.360	4.481E-03	11.498	9.608E-04	11.605	9.094E-04
Superior frontal gyrus, medial orbital	ORBsupmed.L	13.738	3.073E-04	17.707	5.189E-05	15.983	1.138E-04
Superior frontal gyrus, medial orbital	ORBsupmed.R	9.460	2.550E-03	11.364	1.025E-03	9.933	2.074E-03
Gyrus rectus	REC.L	10.414	1.575E-03	12.878	4.929E-04	11.018	1.212E-03
Gyrus rectus	REC.R	11.841	7.745E-04	14.951	1.848E-04	15.564	1.382E-04
Insula	INS.R	16.762	7.314E-05	20.292	1.628E-05	19.584	2.216E-05
Anterior cingulate and paracingulate gyri	ACG.L	23.808	2.994E-06	31.936	1.210E-07	30.798	1.888E-07
Anterior cingulate and paracingulate gyri	ACG.R	20.112	1.563E-05	26.499	1.124E-06	29.749	2.893E-07
Hippocampus	HIP.L	9.356	2.688E-03	11.857	8.065E-04	11.279	1.066E-03
Parahippocampal gyrus	PHG.L	9.929	2.010E-03	10.207	1.814E-03	9.091	3.166E-03
Amygdala	AMYG.L	6.928	9.489E-03	9.037	3.261E-03	8.824	3.627E-03
Amygdala	AMYG.R	6.130	1.454E-02	9.640	2.406E-03	9.287	2.869E-03
Fusiform gyrus	FFG.L	12.760	4.937E-04	18.645	3.396E-05	16.192	1.033E-04
Superior parietal gyrus	SPG.R	6.060	1.511E-02	9.433	2.671E-03	8.274	4.800E-03
Inferior parietal, but supramarginal and angular gyri	IPL.R	14.144	2.528E-04	14.631	2.147E-04	13.377	3.871E-04
Supramarginal gyrus	SMG.R	9.648	2.318E-03	8.856	3.573E-03	8.098	5.255E-03
Lenticular nucleus, putamen	PUT.R	6.381	1.271E-02	9.017	3.293E-03	7.658	6.596E-03
Lenticular nucleus, pallidum	PAL.L	7.506	6.995E-03	9.399	2.716E-03	7.747	6.300E-03
Heschl gyrus	HES.L	4.511	3.554E-02	9.418	2.691E-03	6.794	1.037E-02
Heschl gyrus	HES.R	3.751	5.490E-02	6.841	1.012E-02	7.814	6.085E-03
Superior temporal gyrus	STG.L	12.718	5.041E-04	20.726	1.344E-05	18.400	3.769E-05
Temporal pole: superior temporal gyrus	TPOsup.L	19.497	2.067E-05	22.744	5.566E-06	19.856	1.962E-05
Temporal pole: superior temporal gyrus	TPOsup.R	8.458	4.260E-03	12.277	6.581E-04	15.092	1.722E-04
Middle temporal gyrus	MTG.L	8.927	3.348E-03	15.769	1.263E-04	13.292	4.031E-04
Temporal pole: middle temporal gyrus	TPOmid.L	5.247	2.356E-02	6.895	9.840E-03	8.588	4.090E-03
Temporal pole: middle temporal gyrus	TPOmid.R	6.494	1.196E-02	8.231	4.915E-03	7.514	7.109E-03
Inferior temporal gyrus	ITG.L	11.019	1.164E-03	17.948	4.652E-05	14.251	2.557E-04

Supplementary Table 3. Information on brain regions in which nodal efficiency was significantly reduced by PDE4D variants in the NC group.

NC		rs2409627		rs9647533		rs11955845	
Regions	abbr.	F	P	F	P	F	P
Precentral_R	PreCG.R	4.41	4.25E-02	4.41	4.25E-02	4.41	4.25E-02
Rolandic_Oper_R	ROL.R	5.25	2.77E-02	5.25	2.77E-02	5.25	2.77E-02
Olfactory_L	OLF.L	15.33	3.73E-04	15.33	3.73E-04	15.33	3.73E-04
Frontal_Mid_Orb_L	ORBsupmed.L	6.79	1.31E-02	6.79	1.31E-02	6.79	1.31E-02
Frontal_Mid_Orb_R	ORBsupmed.R	5.48	2.48E-02	5.48	2.48E-02	5.48	2.48E-02
Rectus_L	REC.L	15.60	3.38E-04	15.60	3.38E-04	15.60	3.38E-04
Rectus_R	REC.R	18.68	1.12E-04	18.68	1.12E-04	18.68	1.12E-04
Cingulum_Ant_L	ACG.L	18.67	1.12E-04	18.67	1.12E-04	18.67	1.12E-04
Cingulum_Ant_R	ACG.R	21.90	3.78E-05	21.90	3.78E-05	21.90	3.78E-05
Hippocampus_L	HIP.L	15.76	3.18E-04	15.76	3.18E-04	15.76	3.18E-04

Supplementary Table 4. Information on brain regions in which nodal efficiency was significantly reduced by PDE4D variants in the MCI group.

MCI		rs2409627		rs9647533		rs11955845	
Regions	abbr.	F	*P*	F	*P*	F	*P*
Rolandic_Oper_R	ROL.R	7.72	7.30E-03	7.25	9.16E-03	7.25	9.16E-03
Supp_Motor_Area_L	SMA.L	8.76	4.40E-03	10.38	2.06E-03	10.38	2.06E-03
Supp_Motor_Area_R	SMA.R	6.37	1.43E-02	7.37	8.67E-03	7.37	8.67E-03
Cingulum_Ant_L	ACG.L	3.20	7.88E-02	4.68	3.45E-02	4.68	3.45E-02
Fusiform_L	FFG.L	5.97	1.75E-02	5.61	2.10E-02	5.61	2.10E-02
Temporal_Sup_L	STG.L	8.56	4.85E-03	8.44	5.13E-03	8.44	5.13E-03
Temporal_Pole_Sup_L	TPOsup.L	9.85	2.63E-03	8.34	5.37E-03	8.34	5.37E-03
Temporal_Mid_L	MTG.L	8.00	6.34E-03	7.24	9.21E-03	7.24	9.21E-03

Supplementary Table 5. Information on brain regions in which nodal efficiency was significantly reduced by PDE4D variants in the AD group.

AD		rs2409627		rs9647533		rs11955845	
Regions	abbr.	F	*P*	F	*P*	F	*P*
Middle frontal gyrus	MFG.R	2.784	1.072E-01	9.065	1.964E-02	9.065	1.964E-02
Inferior frontal gyrus, orbital part	ORBinf.L	3.244	8.329E-02	8.955	2.015E-02	8.955	2.015E-02
Rolandic operculum	ROL.R	3.131	8.857E-02	7.959	2.573E-02	7.959	2.573E-02
Olfactory cortex	OLF.L	3.251	8.299E-02	20.701	2.636E-03	20.701	2.636E-03
Olfactory cortex	OLF.R	0.560	4.610E-01	7.965	2.569E-02	7.965	2.569E-02
Anterior cingulate and paracingulate gyri	ACG.L	3.353	7.856E-02	13.639	7.725E-03	13.639	7.725E-03
Anterior cingulate and paracingulate gyri	ACG.R	2.381	1.349E-01	9.923	1.615E-02	9.923	1.615E-02
Superior temporal gyrus	STG.L	2.189	1.510E-01	9.153	1.924E-02	9.153	1.924E-02
Middle temporal gyrus	MTG.L	1.677	2.067E-01	9.684	1.704E-02	9.684	1.704E-02
Inferior temporal gyrus	ITG.L	0.910	3.490E-01	6.782	3.520E-02	6.782	3.520E-02

Supplementary Table 6. Number of subjects with different genotypes in each diagnostic group.

Subjects	rs2409627		rs9647533		rs11955845	
	Wild-type	Variants	Wild-type	Variants	Wild-type	Variants
NC (n=42)	31	11	31	11	31	11
MCI (n=65)	51	14	51	14	50	15
AD (n=30)	22	8	22	8	23	7
